# 한국어판 집중치료 후 증후군 지식 측정도구의 문화적 적응 및 타당도와 신뢰도: 방법론적 연구

**DOI:** 10.7586/jkbns.26.027

**Published:** 2026-08-27

**Authors:** Jiyoon Kang, Hyosung Cha

**Affiliations:** 1 Seoul National University Bundang Hospital, Seongnam, Korea; 1 분당서울대학교병원; 2 College of Nursing, Korea University, Seoul, Korea; 2 고려대학교 간호대학; 3 College of Nursing, Eulji University Seongnam Campus, Seongnam, Korea; 3 을지대학교 간호대학

**Keywords:** Postintensive care syndrome, Knowledge, Surveys and questionnaires, Psychometrics, 집중치료 후 증후군, 지식, 설문 및 질문지, 심리측정학

## Abstract

**Purpose:**

This study aimed to develop the Korean version of the Post-Intensive Care Syndrome Knowledge Test (K-PICS-KT) through cross-cultural adaptation to ensure cultural and clinical appropriateness for use in Korea and to evaluate its validity and reliability.

**Methods:**

The Post-Intensive Care Syndrome Knowledge Test (PICS-KT) was translated into Korean through a cross-cultural adaptation process based on the World Health Organization guidelines for instrument translation and adaptation, with consideration of the Korean cultural and clinical context. The preliminary K-PICS-KT was then evaluated for validity and reliability. Validity evidence was examined using known-groups validity and item analysis. Reliability was evaluated by assessing internal consistency with the Kuder–Richardson Formula 20 (KR-20) and temporal stability with a test–retest design.

**Results:**

Content validity assessment produced a preliminary K-PICS-KT comprising 54 items across four domains. Known-groups validity was supported (p < .001). During item analysis, one item was removed because of a low item–total correlation and content review, resulting in a final K-PICS-KT comprising 53 items across four domains. The KR-20 coefficient was .97, and the test–retest intraclass correlation coefficient was .94. Evidence supporting content validity, known-groups validity, item discrimination, internal consistency reliability, and test–retest reliability was established.

**Conclusion:**

The K-PICS-KT developed in this study can be used to measure PICS knowledge among intensive care unit nurses, develop educational programs to improve knowledge, and evaluate the effectiveness of PICS education programs.

## 서론

### 1. 연구의 필요성

의료기술의 발달과 중환자 진료체계 향상으로 중환자실 환자의 생존율이 증가하면서[1], 집중치료 후 증후군(post-intensive care syndrome, PICS)도 증가하고 있다[2]. PICS는 2010년 미국중환자의학회에서 제시된 개념으로 중환자실 치료 이후 새롭게 발생하거나 기존 상태가 악화된 신체적·인지적·정신적 건강 문제를 의미한다[3]. PICS의 주요 위험요인으로 고령, 여성, 동반된 정신질환, 섬망, 질병 중증도, 부동 상태, 장기간 인공호흡기 사용, 중환자실 장기 재원, 패혈증, 진정제 사용, 중환자실에서의 부정적인 경험 등이 보고되고 있다[4,5]. 예방 및 치료를 위한 중재로는 ABCDEF bundle 적용이 핵심 중환자 간호 전략으로 강조되고 있으며, 적절한 영양공급, 중환자실 다이어리 작성 등이 권고되고 있다[4].

중환자실 생존자의 50%~70%에서 PICS와 관련된 다양한 신체적·인지적·정신적 건강 문제가 나타나고 있으며[4,6], 증상은 퇴원 후 3개월까지 악화되다가 6개월, 12개월이 지나면서 호전되는 경향을 보이지만 입원 전 건강 상태로 완전히 회복되지 못하고[7], 퇴원 후 5~15년까지 지속되기도 한다[8]. 또한 중환자실 생존자의 퇴원 3개월 이후 직장 복귀율은 35%에 그쳐, 사회로의 복귀에 어려움이 있으며[9] 지속적인 의료 이용으로 인해 경제적 부담이 증가하기도 한다[10]. 뿐만 아니라, PICS는 생존자 가족에게도 수면장애, 불안, 우울, 외상 후 스트레스 장애와 같은 심리적 문제를 초래하여 삶의 질을 저하시킬 수 있으므로[4,11,12], 중환자실 입원 단계에서부터 예방 및 치료를 위한 적극적인 중재가 요구된다[13]. 그러나 PICS는 명확한 원인이나 정의된 특징이 없이 복합적인 증상들로 이루어진 증후군으로[14], 위험요인과 증상의 다양성, 다차원적인 특성으로 인해 이해에 어려움을 줄 수 있다. 의료진의 PICS 지식과 인식 수준은 전반적으로 낮은 것으로 보고되었는데, 독일어권의 소아중환자실 의료진 대상 연구에서 31%만이 PICS를 임상적으로 중요한 개념으로 인식하고 있었고, 사후관리의 어려움으로는 필요성에 대한 인식 부족을 보고하였다[15]. 미국의 중환자실 의료진 대상 연구에서는 PICS의 위험요인과 증상에 대한 지식이 낮게 나타났다[14]. 국내 성인중환자실 간호사 대상 연구에서 69.7%가 PICS 용어를 들어본 적이 없었으며, 5.7%만이 중요한 용어라고 답하여 PICS에 대한 의료진의 인식이 전반적으로 낮은 것으로 나타났다[16]. 중환자실 간호사는 PICS 예방 중재 제공에 있어 핵심적인 역할을 담당하기 때문에[17], PICS에 대한 충분한 지식을 갖추고 PICS 예방을 위해 통증 및 섬망의 정기적인 평가와 예방, 진정제 및 진통제 사용의 최소화, 조기 재활 및 운동, 적절한 영양공급, 환자 가족의 참여를 촉진하는 근거기반 간호중재를 체계적으로 수행해야 한다[13].

지금까지 국내외 의료진의 PICS 지식 및 인식을 확인하기 위해 사용된 도구는 체계적인 타당도와 신뢰도 검증을 거친 도구가 아닌, 문헌 검색과 내용타당도를 기반으로 만들어진 도구를 사용하거나[14,15] 한글로 번안 후 내용타당도만 확인한 도구가 사용되고 있다[16]. 특히 국내의 성인중환자실 간호사를 대상으로 한 연구에서 사용된 도구는 PICS 관련 항목 중 가장 중요하다고 생각하는 하나를 선택하도록 하거나 PICS 중재 수행 여부를 확인하는 방식으로 구성되어 있어[16], PICS의 전반적인 지식을 포괄적으로 평가하기에는 제한적이다.

Sürmeli 등[18]은 의료진의 PICS 지식 정도를 측정하기 위해 타당도와 신뢰도가 검증된 집중치료 후 지식 측정도구(Post-Intensive Care Syndrome Knowledge Test, PICS-KT)를 개발하였다. PICS-KT는 일반적 사항 4문항, 위험요인/원인 16문항, 징후/증상 10문항, 예방/치료 중재 16문항의 총 4개 영역과 46문항으로 구성되어 있으며, 자가보고식 설문 형식으로 ‘Yes’ 또는 ‘No’ 중 하나를 선택하도록 구성되어 있다. 정답은 1점, 오답은 0점을 부여하여 총점의 범위는 0점에서 46점이다[18]. 그러나 해당 도구는 국내 임상환경과 문화적 맥락을 충분히 반영하지 못하는 한계가 있다.

이에 본 연구는 Sürmeli 등[18]이 개발한 PICS-KT를 한국의 문화적 상황 및 임상환경을 반영하여, 문화적 적응(cross-cultural adaptation)을 포함한 한국어판 집중치료 후 증후군 지식 측정도구(Korean version of Post-Intensive Care Syndrome Knowledge Test, K-PICS-KT)를 개발한 후 타당도와 신뢰도를 검증하고자 한다. 이를 통해 K-PICS-KT를 중환자실 간호사의 PICS 지식 평가 도구로 활용하여 향후 PICS 교육 프로그램의 효과를 평가하고, PICS 예방 및 중재 전략 수립에 기여하고자 한다.

### 2. 연구의 목적

본 연구의 목적은 Sürmeli 등[18]의 PICS-KT를 한국의 문화적 상황 및 임상환경에 적합하도록 문화적 적응을 포함한 한국어 번안 과정을 거쳐 K-PICS-KT를 개발하고 타당도와 신뢰도를 검증하는 것이다.

## 연구 방법

### 1. 연구 설계

본 연구는 의료진의 집중치료 후 증후군 지식 측정도구인 PICS-KT [18]를 한국의 문화적 상황 및 임상적 특성을 고려하여 문화적 적응 과정을 거쳐 한국어로 번안하고, K-PICS-KT를 개발 후 타당도와 신뢰도를 검증하기 위한 방법론적 연구이다.

### 2. 연구 대상

상급종합병원 성인중환자실 간호사를 대상으로 본 연구를 이해하고 자발적으로 연구 참여에 동의한 자로 편의표집 하였다. 본 연구의 표본크기는 G*Power 3.1.9.7 프로그램을 이용하여 산출하였다. 타당도 검증을 위한 독립적 두 집단간 비교를 위해 independent t-test를 기준으로 선행연구[18]에 근거하여 중간 효과크기(d) .64, 유의수준(*α*) .05, 검정력(1-*β*) .95를 고려하여 집단별 최소 표본 수 65명이 산출되었다. 탈락률을 고려하여 집단별 77명 총 154명을 목표로 하였으며, 불충분한 응답을 제외한 최종 145명의 자료를 분석에 활용하였다. 이는 문항분석을 위해 최소 100명의 표본이 필요하다는 권고 기준[19]을 만족하였다.

### 3. 연구 절차

#### 1) 문화적 적응 및 한국어 번안

PICS-KT [18] 개발자인 Sürmeli로부터 도구 사용 및 수정에 대한 승인을 받은 후 세계보건기구(World Health Organization, WHO)의 번역 지침[20]을 근거로 한국의 문화적, 임상적 상황을 고려한 순번역, 번역본 통합, 역번역, 전문가 위원회 검토를 거쳐 PICS-KT의 문화적 적응을 포함한 한국어 번안을 시행하였다.

첫 번째, 순번역은 한국어와 영어를 유창하게 사용하는 번역자 2인에 의해 시행되었다. 번역자 2인은 모두 중환자실 간호사 경력이 있으며 미국에서 간호학 교수와 한국의 상급종합병원 중환자실 전문간호사로 재직 중으로 PICS에 대한 지식을 가지고 있다. 번역자 2인은 독립적으로 한국의 문화적 상황 및 간호실무 상황을 고려하여 영문에서 한글로 PICS-KT를 번역하였고, 이후 2인의 번역 내용을 본 연구의 책임연구자가 통합하였으며 통합본은 번역자 2인에 의해 검토 및 확인하였다. 이 과정에서 한국어만으로 이해하기 어려운 단어는 임상실무에서 흔히 사용되는 의학용어의 영어를 함께 적어주거나, 단어의 의미를 함께 적어 이해하기 쉽도록 하였다. ‘muscle weakness’를 ‘근육 쇠약’과 ‘근육 위약’으로 각각 번역했던 것을 ‘근력 저하(muscle weakness)’로 변경하였고, ‘mental fogginess’를 ‘정신적 혼탁’과 ‘정신적 혼미’로 각각 번역했던 것을 ‘정신적 흐림(mental fogginess: 사고의 속도 저하, 집중력 저하, 기억력 감퇴 등 주관적 인지 기능 저하 상태)’으로 수정하였다.

두 번째, PICS에 대한 지식이 없으며 의료인이 아닌 2인의 번역가가 한글에서 영어로 역번역하였다. 역번역자 1인은 영어가 모국어이면서 한국어에 능통한 번역자이고, 또 다른 역번역자는 한국어를 모국어로 사용하지만 20년 이상 영어권에서 거주하며 영어와 한국어에 모두 능통한 번역자이다. 역번역자 2인은 PICS-KT 원도구에 대한 정보 없이 한글로 번역된 통합본을 영어로 독립적으로 역번역하였다.

세 번째, 6인의 전문가로 구성된 전문가 위원회의 검토가 시행되었다. 전문가 위원회는 성인중환자실 책임간호사 2인, 영어권에서 간호사로 근무한 경험이 있어 영어와 한국어에 모두 능통하고 중환자실 경력이 있는 간호사 2인, 도구개발의 경험이 있는 간호학과 교수 1인, 중환자실 간호사 20년 경력과 중환자전문간호사 자격을 보유한 연구자 1인으로 구성되었으며 PICS-KT 원도구와 번역본, 번역통합본, 역번역본을 통합적으로 검토하였다. 이 과정에서 ‘ICU diary’를 한글로 번역한 ‘중환자실 일기’는 ‘중환자실 간호일지’와 혼동이 있다는 의견에 따라 한글과 영문을 혼용하여 ‘중환자실 다이어리’로 번안하였다. 또한 ‘중환자실 다이어리’와 ‘동료지지’에 대한 단어의 이해도를 높이기 위해 추가 설명을 함께 표기하여 ‘중환자실 다이어리(중환자실 입실 중 의료진과 가족이 환자에게 일어나는 일에 대해 적는 일기 형식)’[13], ‘동료지지(중환자실 경험을 한 환자 간 소통, 공감 및 조언 제공을 포함하는 활동)’[21]로 수정하였고, 가독성을 위해 ‘집중치료 후 증후군’ 문구를 모두 PICS로 수정하였다.

전문가 위원회 검토에 의해 수정된 도구는 번역가와 역번역가에 의해 추가 검토 및 확인하였으며 이 과정에서 ‘정신적 흐림(mental fogginess)’은 같은 의미로 더 널리 사용되고 있는 ‘브레인 포그(brain fog)’로 변경하는 것이 적절하다는 의견에 따라, 도구의 개발자에게 ‘mental fogginess’를 ‘brain fog’로 변경하는 것이 의미 변화가 없고 적절한지 확인하는 과정을 거친 후 ‘브레인 포그(brain fog: 주의력 저하, 기억력 저하, 사고력 저하 등 인지 기능 저하 상태)’[22]로 변경하였다.

#### 2) 내용타당도 검증

내용타당도 검증을 위해 7인을 대상으로 전문가 내용타당도를 평가하였다[23]. 전문가 구성은 중환자 실무교수를 겸임하고 있는 호흡기내과 의사 1인과 외과 의사 1인, 중환자실 20년 경력의 중환자실 수간호사 1인, 중환자실 경력 각각 10년, 18년의 중환자실 전문간호사 2인, 중환자실 경력 16년의 중환자실 책임간호사 2인으로 구성하였다. 2차에 걸친 내용타당도 검증을 위해 동일한 전문가에게 2주 간격으로 내용타당도 검증을 시행하였으며[23], 그 결과 총 54문항의 K-PICS-KT 예비문항이 도출되었다.

#### 3) 예비조사

K-PICS-KT 예비문항의 이해도 확인을 위해, 중환자실 간호사 10명을 대상으로 예비조사를 시행하였다. 예비조사 참여자의 평균 연령은 27.80 ± 2.66세, 중환자실 경력은 4.53 ± 2.39년이었으며, 모두 PICS 교육을 받은 적이 없었다. K-PICS-KT의 각 문항의 이해도를 1점 ‘매우 이해하기 어렵다’, 2점 ‘이해하기 어렵다’, 3점 ‘이해하기 쉽다’, 4점 ‘매우 이해하기 쉽다’의 4점 척도로 평가한 결과 각 문항의 이해도는 3.8~3.9점, 평균 이해도는 3.88 ± 0.30점이었다. 설문조사 소요시간은 평균 5.30 ± 1.25분이었으며 예비조사 후 모든 참여자를 대상으로 문항의 이해도와 수정 필요 여부를 확인하였다. 모든 참여자는 K-PICS-KT의 54문항을 이해하는 데 어려움이 없었으며, 수정이 필요하다는 의견도 없었다. 이에 따라 54문항의 이해도가 양호함을 확인한 후 별도의 수정 없이 K-PICS-KT 예비도구를 완성하였다.

### 4. 자료 수집

자료 수집은 2026년 2월 27일부터 4월 1일까지 온라인 설문조사를 통해 실시하였다. 분당서울대학교병원 간호본부의 승인을 받은 후 공고문을 게시하였으며 연구 참여자 모집 공고문의 QR코드 또는 링크를 통해 연구 설명문과 참여 동의 안내문을 읽고, 자율적 참여를 통해 온라인 설문조사가 시행되었다. 검사-재검사 신뢰도 검증을 위해 동의한 대상자에게 첫번째 설문조사 2주 후 설문 링크를 발송하여 K-PICS-KT 예비도구의 설문조사를 재시행하였다.

### 5. 자료 분석

수집된 자료는 SPSS 25.0(IBM Corp. Armonk, NY, USA)로 분석하였으며, 통계적 검정은 양측검정으로 유의수준 .05로 설정하였다. 대상자의 일반적 특성은 기술통계와 빈도분석을 사용하였다. 도구의 타당도 검증을 위해 첫째, 전문가 집단의 item-level content validity index (I-CVI), scale-level content validity index (S-CVI), S-CVI/UA (universal agreement), S-CVI/Ave (average)를 산출하여 내용타당도를 분석하였고 둘째, 집단비교법과 문항분석을 시행하여 타당도를 검증하고자 하였다. 또한 신뢰도 검증을 위해 KR-20을 이용한 내적일관성, 검사-재검사 신뢰도를 이용한 시간적 안정성을 검증하고자 하였다.

타당도 검증을 위해 PICS에 대해 들어본 경험이 있는 집단이 그렇지 않은 집단보다 K-PICS-KT 점수가 높을 것이라는 사전가설을 설정하였으며, 집단비교법을 이용한 independent t-test를 통해 분석하였다. 문항분석은 고전검사이론에 근거를 두고 문항 난이도(item difficulty indexes), 변별도(discrimination indexes), 문항-총점 상관계수를 확인하였다. 문항 난이도는 각 항목에서 응답자 수에 대한 정답률로 제시하고, 문항 변별도는 Kelly (1939)[24]에 근거하여 대상자의 총점을 성적순으로 배열하고 상위 27%와 하위 27%를 각각 상위집단과 하위집단으로 나누어 계산하는 변별도 지수와 각각의 문항과 총점 간의 상관계수를 도출하는 문항-총점 상관계수(corrected item–total correlation)를 산출하였다. 도구의 신뢰도 검증을 위해 첫째, 이분형 문항의 내적일관성 검증을 위한 Kuder-Richardson Formula 20 (KR-20)값을 산출하였으며, 문항 삭제 시 KR-20 값의 변화를 확인하였다. 둘째, 안정성 신뢰도 검증을 위해 검사-재검사 신뢰도는 Intraclass Correlation Coefficient (ICC)를 이용하여 산출하였다.

### 6. 윤리적 고려

본 연구는 연구자가 속한 기관의 Institutional Review Board (IRB) 승인(IRB No. B-2509-996-301)을 받은 후 관련 기준에 따라 수행되었으며, 승인 이후 자료수집이 이루어졌다. 연구 참여자 모집 공고문을 확인 후 연구 참여에 자발적으로 동의한 간호사가 링크에 접속하면 연구의 목적, 방법, 자료수집방법과 소요시간, 연구 참여와 철회의 자율성, 연구 참여 철회에 따른 불이익 없음, 수집된 자료의 비밀 유지 등에 관한 사항을 한번 더 안내하였다. 연구 참여 동의 화면에서 동의 여부를 확인하고, 동의하는 경우에만 설문문항으로 넘어가도록 하여 설문조사를 시행하였다. 연구 참여자에게는 감사의 의미로 소정의 답례품을 제공하였다.

## 연구 결과

### 1. 대상자의 일반적 특성

본 연구 대상자의 평균 연령은 30.74 ± 5.03세였고, 여성이 119명(82.1%)이었으며 총 임상경력은 평균 6.68 ± 4.88년, 중환자실 경력은 평균 5.66 ± 4.21년으로 나타났다. 중환자실 근무부서는 내과계가 51명(35.2%)으로 가장 많았으며, 직위는 일반간호사가 102명(70.3%), 최종 학력은 학사가 115명(79.3%)으로 가장 많았다. PICS에 대해 들어본 적이 있다고 응답한 대상자가 76명(52.4%)이었으며, PICS 교육 경험이 있는 대상자는 20명(13.8%)이었다(Table 1).

### 2. 타당도 검증

#### 1) 내용타당도

PICS-KT를 한국어로 번안한 46문항에 대한 1차 전문가 내용타당도 검증에서 I-CVI는 .71~1.00이었고, S-CVI는 S-CVI/UA와 S-CVI/Ave가 모두 .94로 각각 나타났다. I-CVI가 .71인 3개 문항은 전문가 의견과 연구팀의 검토를 거쳐, 다음과 같이 1개를 삭제하고 2개를 수정하였다. ‘PICS는 중환자실 퇴실을 원하지 않는 환자들이 겪게 되는 증후군이다.’ 문항은 잘못된 정의의 예시가 개연성이 낮으며 PICS 지식을 평가하는 목적에 부합하지 않다는 중환자실 담당 전문의 의견에 의해 삭제하고, ‘집중력 문제는 PICS 증상이다.’ 문항은 ‘집중력 저하는 PICS 증상이다.’로 수정하였으며, ‘중환자실 환자의 조기 이상 지연은 PICS를 예방할 수 있는 중재 중 하나이다.’ 문항은 ‘조기 이상 지연’이 흔히 사용되지 않아 이해하기 어렵다는 의견에 의해 ‘중환자실 환자가 늦게 거동을 시작하는 것은 PICS를 예방할 수 있는 중재 중 하나이다.’로 변경하였다. 또한 전문가의 의견에 따라 PICS의 일반적 사항의 3문항 ‘PICS 환자는 신체적 회복 이후에도 원래의 직업이나 일상생활로 복귀하는 데 어려움을 겪을 수 있다.’, ‘섬망은 PICS와 동일한 질환이다’, ‘PICS는 중환자실 퇴실 후 단기간에 자연 소실되는 경우가 대부분이다’, 징후/증상 영역의 3문항 ‘PICS 증상은 중환자실 퇴실 후 수개월에서 수년까지 지속될 수 있다’, ‘중환자실 환자의 가족이 느끼는 죄책감이나 분노는 집중치료 후 가족 증후군(post-intensive care syndrome-family, PICS-F)의 주요 증상 중 하나이다’, ‘중환자실 환자의 가족은 환자 퇴원 후에도 불면증, 식욕 부진, 극심한 피로감을 느낄 수 있다’, 예방/치료 중재 영역의 3문항 ‘소음 감소, 조명 조절, 시계와 달력을 이용한 지남력 제공은 중환자실의 환경적 스트레스를 줄이고 PICS를 예방할 수 있는 중재 중 하나이다’, ‘PICS 예방과 관리를 위해 다학제적 협력(의사, 간호사, 재활치료사, 영양사, 정신건강 전문가 등)이 중요하다’, ‘중환자실 퇴실 후 PICS에 대한 추가적인 관리가 필요하지 않다’를 추가하였다. 총 54문항으로 수정된 K-PICS-KT에 대해 2차 내용타당도 검증을 실시하였으며, 추가된 문항의 요인 적합성도 '전혀 적합하지 않다’ 1점에서 ‘매우 적합하다’ 4점으로 평가하였다. 2차 전문가 내용타당도 검증 결과, 54문항에 대한 I-CVI 값이 .857~1.00, S-CVI/UA 값이 .981, S-CVI/Ave 값이 .997로 나타났으며, 추가된 모든 문항은 해당 요인에 적합한 것으로 확인되었다[25].

#### 2) 집단비교법, 문항분석

타당도 검증을 위해 집단비교법과 문항분석을 이용하였다. 집단간 비교를 위해 Sürmeli 등[18]을 토대로 이전에 PICS에 대해 들어본 경험의 유무에 따라 두 집단을 비교하였다. 그 결과 PICS에 대해 들어본 적이 있는 집단이 그렇지 않은 집단에 비해 K-PICS-KT 점수가 유의하게 높았으며(t = 4.03, *p* < .001, Cohen's d = 0.69, mean difference = 9.25, 95% confidence interval [CI]: 4.69–13.81), 이를 통해 집단비교법을 이용한 기준집단 타당도에 대한 일부 근거를 확인하였다(Table 2).

문항분석은 고전검사이론에 근거하여 문항 난이도(item difficulty indexes, IDI), 변별도(discrimination indexes, DI), 문항-총점 상관계수를 이용하였다. K-PICS-KT의 IDI 범위는 .32~.91로 평균 .75이었다(Table 3). 54문항 중 23문항(42.6%)은 난이도가 .80 이상으로 쉬운 문항이었으며, 31문항(57.4%)은 난이도가 .30~.80 미만으로 적절한 문항이었고, 어려운 문항은 없는 것으로 나타났다[25]. 가장 쉬운 문항은 8번, 43번, 46번, 53번 문항이었다. K-PICS-KT의 DI 범위는 .30~.79이며, 평균은 .50이었다(Table 3). 고전검사이론을 적용한 경우, 변별도 지수가 .40 이상은 변별력이 높은 문항, .21~.40 미만은 변별력이 있는 문항 .20 미만은 변별력이 낮은 문항으로 해석하는데[26], 54문항 중 42문항(77.8%)은 변별력이 높은 문항, 12문항(22.2%)은 변별력이 있는 문항으로 확인되었다. 문항-총점 상관계수의 범위는 .26~.87이며, 평균은 .64였다(Table 3). 문항-총점 상관계수가 .30 미만이면 변별력이 낮은 문항으로 해석하는데[27] 1개 문항의 문항-총점 상관계수가 .26으로 확인되었다. 이에 PICS 관련 선행연구를 검토하고 전문가 집단과 원도구 개발자의 의견을 종합적으로 고려한 결과, 해당 문항인 ‘비만은 PICS 발생 위험을 높일 수 있다’를 최종 삭제하였다.

### 3. 신뢰도 검증

신뢰도 검증을 위해 내적일관성 신뢰도와 시간적 안정성 신뢰도를 확인하였다. 54개 예비문항에 대한 내적일관성 신뢰도 검증 결과, KR-20 값은 .97이었으며, 19번 문항 삭제 후 최종 53개 문항의 KR-20 값은 .97로 나타났다(Table 3). 시간적 안정성 신뢰도 검증을 위한 재검사에는 추가 설문조사에 동의한 133명 중 37명(27.8%)이 참여하였다. 시간적 안정성 신뢰도 검증을 위한 재검사 대상자 37명의 평균 연령은 30.57 ± 3.72세, 총 임상경력은 평균 6.91 ± 3.86년, 중환자실 경력은 평균 6.27 ± 3.65년이었으며, 재검사에 참여한 군과 참여하지 않은 군 간의 평균 연령(t = 0.25, p = .805), 총 임상경력(t = −0.33, *p* = .743), 중환자실 경력(t = −1.01, *p* = .314)에서 통계적으로 유의한 차이는 없었다. 검사-재검사 간의 ICC 값은 .94 (95% CI: 0.88–0.97)로 나타나(Table 4), K-PICS-KT의 검사-재검사 신뢰도에 대한 근거를 확보하였다[28].

### 4. 최종 도구 개발

K-PICS-KT는 ‘일반적 사항’ 6문항, ‘위험요인/원인’ 15문항과 ‘징후/증상’ 15문항, ‘예방/치료 중재’ 17문항으로 총 53개 문항, 4개 영역으로 이루어져 있다(Appendix 1). 문항은 ‘맞다’, ‘틀리다’, ‘모른다’ 중 하나를 선택하도록 구성된 자가보고식 설문 형식이다. 정답이면 1점, 오답 또는 ‘모른다’를 선택하면 0점을 부여하며, 총점의 범위는 0점에서 53점이다. 점수가 높을수록 PICS에 대한 지식이 높음을 의미한다. 최종 도구에서 ‘맞다’가 정답인 항목은 1번, 2번, 3번, 4번, 7번, 8번, 10번, 13번, 16번, 19번, 20번, 23번, 25번, 26번, 28번, 29번, 31번, 34번, 35번, 36번, 38번, 41번, 42번, 43번, 45번, 46번, 48번, 50번, 52번으로 총 29문항이며, ‘틀리다’가 정답인 항목은 나머지 24문항이다.

## 논의

본 연구는 Sürmeli 등[18]의 PICS-KT를 WHO 번역 지침[20]에 근거하여 한국의 문화적 상황과 임상적 특성을 반영하여 문화적 적응을 포함한 한국어 번안 과정을 거쳐 K-PICS-KT를 개발하고, 성인중환자실 간호사를 대상으로 타당도와 신뢰도를 검증하고자 수행되었다.

문화적 적응을 위해, 한국의 임상현장에서 오해 가능성이 있거나 생소하게 받아들여질 수 있는 용어인 중환자실 다이어리(ICU diary), 동료지지(peer support), 브레인 포그(brain fog)를 한국의 임상환경에 맞게 조정하고 필요한 설명을 추가하였다. 이를 통해 원도구의 개념적 동등성을 유지하면서, 문항의 의미를 정확히 이해하도록 하여 도구의 문화적 적합성과 내용의 명확성을 높이고자 하였다. 한편, 역번역자를 제외하고 번역과정에 참여한 번역자 모두가 중환자실 간호 경험과 PICS 지식을 가진 전문가로 구성된 것은 전문적 의미 전달을 위한 측면에서 적절하지만, 비전문가 관점의 이해도를 반영하는 데는 제한이 있을 수 있다.

내용타당도를 평가하기 위한 전문가 패널은 전문간호사뿐만 아니라 중환자 진료를 담당하고 있는 전문의를 포함하여 구성하였다. 이는 PICS가 중환자의학회에서 제시된 개념이라는 점[3]과 PICS 지식 측정도구 개발 과정에서 의학적 배경지식에 대한 전문적인 이해가 함께 요구되는 점을 고려하였다. 또한 중환자 치료에 다학제적 접근의 중요성이 강조되고 있어[29], 중환자 치료에 참여하는 다양한 의료진의 관점을 반영함으로써 도구의 임상 적용 가능성을 높이고자 하였다. 내용타당도 검증 과정에서 ‘일반적 사항’ 3문항, ‘징후/증상’ 3문항, ‘예방/치료 중재’ 3문항의 총 9개 문항이 추가되었다. PICS는 입원 중 예방 및 치료뿐 아니라 퇴원 이후에도 장기적 관점에서의 지속적인 관리가 요구되는데, 이와 관련된 의료진의 지식 부족을 보완하는 것이 중요한 과제로 제시되고 있다[30]. PICS-KT는 PICS 개념을 포함한 일반적 사항과 위험요인, 증상, 예방 및 치료와 관련된 구체적인 지식을 평가하도록 구성되어 있으나, PICS의 추후 관리 및 장기적 관점과 PICS 환자 가족에게 미치는 영향에 대한 내용은 충분히 포함되어 있지 않다. 본 연구에서는 이러한 PICS 지식 측정의 내용적 공백을 보완하고, 한국 임상 상황을 반영하기 위해 관련 문항을 추가하여 도구의 내용적 타당성과 임상적 적용 가능성을 강화하고자 하였다.

먼저, 4개 문항(4,6,34,53번)은 중환자실 퇴실 또는 병원 퇴원 후 PICS 환자의 일상생활 복귀 및 예후, PICS 지속 기간, 퇴실 후 관리의 필요성에 관한 문항으로, ‘일반적 사항’ 2문항과 ‘예방/치료 중재’ 2문항이 각각 추가되었다. 이는 PICS 예방 및 관리가 중환자실 입원 기간 동안에만 초점을 두는 것이 아니라, 환자와 가족의 삶의 질을 포함한 장기적 결과까지 고려한 방향으로 확대되어야 한다는 선행연구[13]를 반영한 결과로 볼 수 있다.

5번 문항은 일반적 사항 영역으로, 섬망과 PICS 개념의 혼동을 감별하기 위한 것이다. 섬망은 PICS의 정신적, 인지적 장애 위험요인이고[31], 섬망 증상은 PICS의 인지적, 정신적 영역과 일부 중복되며, 두 개념 모두 중환자실 입원에서 발생하기 때문에 시간적, 개념적 부분과 관련된 혼동이 있을 수 있다[32]. 이러한 혼동은 PICS 예방과 치료 전략에 불명확성을 초래할 수 있으므로, 두 개념을 명확하게 인식하는 것이 필요하다[32].

다음으로, 집중치료 후 가족 증후군(PICS-F)의 증상과 관련된 2개 문항(35,36번)이 징후/증상 영역에 추가되었다. 한국 사회는 가족 중심 문화가 강하고 가족 구성원 간 긴밀한 상호의존성과 돌봄 책임이 강조되는 문화적 특성이 있다[33]. 이는 한국의 PICS 생존자 가족을 대상으로 PICS-F의 의미를 탐색한 질적 연구[34]에서도 확인되었다. K-PICS-KT는 한국의 문화적 상황을 고려하여, 가족이 경험할 수 있는 PICS-F 증상에 대해서도 의료진의 체계적인 인식과 관리가 필요함이 반영되었다. 또한, 예방/치료 중재 영역에 ‘소음 감소, 조명 조절, 시계와 달력을 이용한 지남력 제공은 중환자실의 환경적 스트레스를 줄이고, PICS를 예방할 수 있는 중재 중 하나이다’[35]라는 46번 문항이 추가되었다. 국내 연구에서 중환자실의 환경적 스트레스가 PICS 관련요인으로 설명되었고[35], 한국의 중환자실 환경이 환자마다 독립된 공간이 아닌 개방형 구조로 이루어진 곳이 대부분이므로, 이러한 임상적 환경을 고려하여 중환자실에서 발생하는 환경적 스트레스를 줄일 수 있는 구체적인 방법[35]이 반영되었다. 이는 환자의 개별적 요구를 존중하고, 중환자실 생존자의 장기적 예후 및 삶의 질 향상에 기여할 수 있는 간호중재로 여겨질 수 있다[36].

마지막으로, PICS 관리를 위한 다학제적 협력의 중요성을 반영하여 예방/치료 중재 영역에 52번 문항이 추가되었는데, PICS의 신체적·인지적·정신적 건강 문제를 다각적인 관점에서 관리하기 위해 다학제적 팀 접근이 필요하며, 다학제적 팀 접근은 중환자실에서뿐 아니라 병원 퇴원 후 추적관찰까지 이어질 수 있도록 해야 한다[4]는 맥락과 동일한 것으로 볼 수 있다. 원도구 개발자로부터 도구의 사용 및 수정에 대한 사전동의를 받은 후 연구를 진행하였으며, 연구 과정에서 주요 용어 변경 및 도구의 구성개념에 영향을 미칠 수 있는 문항의 삭제, 추가 사항을 원도구 개발자에게 다시 공유하여 검토와 최종 확인을 받았다. K-PICS-KT는 원도구보다 문항 수가 7개 많아진 도구이므로 원도구와 총점 및 하위영역 점수를 직접 비교하기에는 한계가 있으며, 구성개념의 범위가 일부 확장되었을 가능성이 있다.

타당도 검증의 집단비교법을 위해 선행연구[18]를 토대로, PICS에 대해 들어본 경험의 유무에 따라 두 집단을 비교하였다. 그 결과, PICS에 대해 들어본 적이 있는 간호사의 K-PICS-KT 점수가 유의하게 높은 것으로 나타나 선행연구의 결과[18]와 일치하였다. 또 하나 주목할 점은 K-PICS-KT 점수가 총 임상경력 및 중환자실 경력과 직위, 교육수준에 따라 유의한 차이를 보이지 않았으나, PICS에 대해 들어본 경험과 PICS 교육 경험에 따라서는 유의한 차이를 보였다는 점이다. 이는 선행연구와 일치하는 결과로[18], K-PICS-KT가 임상경력, 직위와 같은 임상 특성보다는 PICS 관련 경험 및 교육수준에 따른 지식 수준의 차이를 보다 민감하게 반영할 가능성을 의미한다. 따라서 K-PICS-KT는 향후 PICS 관련 경험 및 교육 중재 전후의 지식 수준 변화를 평가하는 데 활용될 수 있을 것으로 사료된다.

본 연구에서는 문항분석 결과, K-PICS-KT는 적절한 난이도 문항이 57.4%, 쉬운 문항이 42.6%이었고, 변별도가 있는 문항이 22.2%, 변별도가 높은 문항이 77.8%로 나타났다. 이는 PICS-KT의 적절한 난이도 문항이54.3%, 쉬운 문항이 45.7%이었고, 변별도가 있는 문항이 26.1%, 변별도가 높은 문항이 73.9%였던 결과와 유사하였다[18]. 특히 추가된 9개 문항 중, 6번 문항을 제외한 모든 문항은 쉬운 난이도이면서 높은 변별도를 보였는데, 이는 해당 문항들이 국내 간호사의 PICS 관련 지식 수준을 구분하는 데 기여할 수 있음을 시사한다. 반면, ‘비만은 PICS 발생 위험을 높일 수 있다’ 문항은 변별력 지수가 .40이었으나, 문항-총점 상관계수가 .30 미만으로 확인되었다. PICS 위험요인에 관한 Gao 등[5]의 연구에 따르면 비만은 확인된 60개의 위험요인에 포함되지만 신체적·인지적·정신적 건강 문제와 사회경제적 영역의 모든 부분에서 효과크기가 큰 주요 위험요인으로 다루어지지 않고 있다. 또한 WHO 기준으로 2022년 비만율을 비교해 보면 PICS-KT 개발 연구[18]가 수행된 튀르키예의 비만율이 34.3%인 것과 달리, 한국의 비만율은 6.7%로 상대적으로 낮은 수준이었다[37]. 이와 같이 문항-총점 상관이 기준보다 낮았고 국내 임상적 맥락과 선행연구 및 전문가와 원도구 개발자의 검토를 종합하여 ‘비만은 PICS 발생 위험을 높일 수 있다’ 문항을 최종 삭제하였다.

신뢰도 검증에 K-PICS-KT의 KR-20 값은 .97로 나타나 높은 내적 일관성을 보였으며, 선행연구[18]의 .93보다 높은 수준이었다. 이는 본 도구가 문항 간 일관성을 바탕으로 PICS 관련 지식을 안정적으로 측정할 수 있음을 시사한다. 다만 매우 높은 신뢰도 값은 문항 간 유사성에 영향을 받을 가능성도 있으므로, 향후 다양한 대상자를 포함한 반복 연구를 통해 도구의 안정성을 지속적으로 확인할 필요가 있다. 시간적 안정성 신뢰도 검증을 위한 검사-재검사에서는 37명의 자료를 분석하였다. 회신율은 28%로 다소 낮았으나, 대상자 수는 도구개발 선행연구[38-40]에서 검사-재검사 신뢰도 검증에 사용된 30~40명과 유사한 수준이었다. 또한 ICC 값은 .94 (95% CI: 0.88–0.97)로 나타나 시간적 안정성에 대한 근거를 확인하였다.

본 연구를 통해 K-PICS-KT는 성인중환자실 간호사의 PICS 지식 수준을 측정하는 데 내용타당도, 기준집단 타당도, 문항분석, 내적일관성 및 검사-재검사 신뢰도에 대한 근거를 확보하였다. K-PICS-KT는 PICS 관련 지식 측정도구를 넘어 PICS 지식을 근거기반 간호실무로 연결하여 교육 및 중환자 간호중재 개발을 위한 기초자료로 활용될 수 있을 것이다. 중환자실 간호사는 PICS를 이해하고 관련된 간호 중재를 적극적으로 수행하며[13], 중환자 간호의 초점을 환자와 가족의 삶의 질 향상을 위한 장기적 예후를 포함하는 방향으로 전환해야 한다[13]. 근거기반의 PICS 예방 간호를 위해 중환자실 다이어리 작성, 조기 재활, 수면 증진은 주요 전략으로 제시되고 있다[13]. 중환자실 다이어리는 중환자실 입실과 퇴실 사이 기억의 간극을 채워주어 환자의 불안과 우울을 감소시키고, 외상 후 스트레스 장애 예방에 도움이 되며[41], 중환자실에서부터 조기 재활은 중환자실 획득 근육쇠약을 줄일 수 있다[13]. 수면증진을 위해 조명 조절 및 귀마개 사용으로 중환자실 환경을 조절하고, 음악요법 중재를 제공하는 것은 중환자실 환자의 수면장애와 불안, 섬망을 줄여줄 수 있다[13]. 이러한 근거를 바탕으로 중환자실 간호사는 PICS 예방을 위한 근거기반 간호중재를 임상실무에 적극적으로 수행하고, PICS 발생 위험을 조기에 발견하는 등의 PICS 예방에서 핵심적인 역할을 수행해야 한다.

본 연구의 제한점으로는 첫째, 연구대상이 특정 기관의 성인중환자실 간호사로 구성되어 있어 다양한 병원 규모, 지역, 중환자실 유형, 직종에 일반화하기에 제한이 있다. 둘째, 타당도 검증이 PICS를 들어본 경험에 따른 집단비교에 제한되어 있어 다양한 가설 검정 또는 외부 준거를 활용한 추가 검증 연구가 필요할 것이다. 또한 타당도 검증을 위한 요인분석이 시행되지 않았으므로, 추후 충분한 대상자 수를 확보한 후 이분형 자료의 요인분석에 적절한 tetrachoric correlation과 WLSMV 기반의 탐색적 요인분석과 확인적 요인분석[42]을 통한 타당도의 추가 검증이 필요할 것이다. 셋째, 검사-재검사 신뢰도에 참여한 대상자는 연령과 경력에 유의한 차이가 없었으나, 대상자 수가 추가 설문조사에 동의한 대상자 중 27.8%만이 참여하였으므로, 재검사 참여자의 대표성이 확보되었다고 보기에는 어려움이 있다. 넷째, 교육 중재 전후 변화에 대한 민감도, 즉 반응도는 아직 검증되지 않았으므로 추후 검증 연구가 필요할 것이다. 그럼에도 불구하고 본 연구는 국내 중환자실 간호사를 대상으로 PICS 지식을 측정할 수 있는 한국어판 도구의 타당도와 신뢰도에 대한 근거를 확인하였으며, 한국의 문화적·임상적 상황을 반영한 문화적 적응 과정을 통해 도구를 개발하였다는 점에서 의의가 있다. 또한 기존 PICS-KT에서 충분히 다루어지지 않았던 PICS 환자의 추후 관리 및 장기적인 맥락에 대해 인식할 수 있도록 K-PICS-KT의 문항을 보완하여, PICS 지식의 포괄적인 측정이 가능하도록 하였다는 점에서도 의의가 있다. K-PICS-KT는 단순한 지식 측정 도구를 넘어, 중환자실 간호사의 PICS 지식 향상 및 근거기반 중환자 간호실무 역량 강화에 기여할 것으로 기대된다.

## 결론

본 연구는 중환자실 간호사를 대상으로 PICS 지식 측정을 위해 문화적 적응을 포함한 한국어판 K-PICS-KT를 개발하고, 타당도와 신뢰도를 검증하였다. 이를 위해 Sürmeli 등[18]의 PICS-KT를 문화적 적응 과정을 거쳐 한국어로 번안하고, 내용타당도와 기준집단 타당도, 문항분석, 내적일관성 및 검사-재검사 신뢰도의 근거를 확보하였다. 최종 확정된 K-PICS-KT는 중환자실 간호사의 PICS 지식 수준을 평가하는 도구로 활용될 수 있으며 PICS 예방 교육과 ABCDEF bundle 관련 간호중재 교육, 중환자실 퇴실 후 관리, PICS-F에 대한 간호사의 인식 향상에도 활용될 수 있을 것이다. 또한 PICS 교육 프로그램의 효과 검증 및 간호사의 지식 수준과 PICS 예방을 위한 임상 수행 간의 관계를 규명하는 연구의 기반을 마련하였다. 이를 통해 K-PICS-KT는 중환자실 간호사의 PICS 관련 역량을 강화하고 근거기반 간호실무 향상에 기여할 것으로 기대된다.

본 연구를 통해 다음을 제언한다. 첫째, 집중치료 후 증후군에 대한 지식 수준을 확인하고, 지식 향상을 위한 교육 프로그램을 개발 및 적용한 후 그 효과를 확인하는 연구를 제언한다. 둘째, 다양한 임상환경과 다양한 직종의 의료진을 대상으로 K-PICS-KT의 타당도와 신뢰도를 검증하는 반복연구를 제언한다.

## Figures and Tables

**Table 1.** General and Clinical Characteristics of Participants (N = 145)

Characteristics	Categories	n (%)	M ± SD
Age (years)			30.74 ± 5.03
Sex	Men	26 (17.9)	
	Women	119 (82.1)	
Total clinical experience (years)			6.68 ± 4.88
ICU clinical experience (years)			5.66 ± 4.21
ICU type	Medical	51 (35.2)	
	Surgical	24 (16.6)	
	Emergency	40 (27.6)	
	Neurology	18 (12.4)	
	Infectious disease	12 (8.2)	
Position	Staff nurse	102 (70.3)	
	Charge nurse	32 (22.1)	
	Advanced practice nurse	7 (4.8)	
	Head nurse	2 (1.4)	
	Nurse educator	2 (1.4)	
Education level	Bachelor’s degree	115 (79.3)	
	Enrolled in a master's program	13 (9.0)	
	Master’s degree	17 (11.7)	
Previously heard of PICS	Yes	76 (52.4)	
	No	69 (47.6)	
PICS education experience	Yes	20 (13.8)	
	No	125 (86.2)	

M = Mean; SD = Standard deviation; ICU = Intensive care unit; PICS = Post-intensive care syndrome.

**Table 2.** Comparison of K-PICS-KT Scores According to General and Clinical Characteristics (N = 145)

Characteristics	Categories	K-PICS-KT	t, F, or r	*p*
		M ± SD		
Age (years)			0.09	.294
Sex	Men	34.85 ± 18.89	-1.84	.076
	Women	41.97 ± 12.51		
Total clinical experience (years)			0.15	.072
ICU clinical experience (years)			0.13	.111
ICU type	Medical	39.98 ± 13.50	0.32	.862
	Surgical	41.38 ± 14.21		
	Emergency	39.45 ± 15.37		
	Neurology	42.78 ± 12.91		
	Infectious disease	43.33 ± 14.80		
Position	Staff nurse	40.52 ± 14.06	0.76	.550
	Charge nurse	39.22 ± 15.87		
	Advanced practice nurse	47.43 ± 3.60		
	Head nurse	51.00 ± 4.24		
	Nurse educator	39.00 ± 1.41		
Education level	Bachelor’s degree	39.63 ± 14.96	1.63	.201
	Enrolled in a master’s program	44.15 ± 12.53		
	Master’s degree	45.24 ± 5.21		
Previously heard of PICS	Yes	45.09 ± 7.09	4.03	< .001
	No	35.84 ± 17.83		
PICS education experience	Yes	45.30 ± 5.74	2.90	.005
	No	39.95 ± 14.85		

K-PICS-KT = Korean version of the Post-Intensive Care Syndrome Knowledge Test; M = Mean; SD = Standard deviation; ICU = Intensive care unit; PICS = Post-intensive care syndrome.

**Table 3.** Item Analysis of the K-PICS-KT (N = 145)

Item No.	Items	IDI	DI	CITC	KR-20 if item deleted (54 item)^†^	KR-20 if item deleted (53 item)^‡^
1	Post-intensive care syndrome (PICS) refers to complications that occur in patients following their experience in the intensive care unit (ICU).	.84	.33	.67	.97	.97
2	PICS can develop even during a patient's ICU stay.	.78	.47	.62	.97	.97
3	PICS can occur among family members of patients admitted to the ICU.	.75	.47	.57	.97	.97
4	Patients with PICS may experience difficulties returning to their previous occupations or daily activities even after physical recovery.	.88	.33	.79	.97	.97
5	Delirium is the same disorder as PICS.	.67	.42	.41	.97	.97
6	PICS usually resolves spontaneously within a short period after ICU discharge.	.46	.30	.31	.97	.97
7	Stress may increase the risk of developing PICS.	.88	.44	.87	.97	.97
8	Patients in the ICU who do not obtain adequate and quality sleep are at risk of developing PICS.	.91	.56	.86	.97	.97
9	ICU patients with prolonged immobility are at low risk of developing PICS.	.74	.58	.59	.97	.97
10	ICU patients with severe infections are at risk of developing PICS.	.83	.44	.73	.97	.97
11	Nutritional status is not a factor affecting the development of PICS.	.74	.40	.65	.97	.97
12	Prolonged sedative use in ICU patients reduces the risk of developing PICS.	.62	.51	.56	.97	.97
13	ICU patients with respiratory failure are at risk of developing PICS.	.82	.58	.73	.97	.97
14	Older ICU patients are at low risk of developing PICS.	.73	.60	.59	.97	.97
15	Pre-existing psychiatric disorders are not risk factors for the development of PICS.	.63	.53	.56	.97	.97
16	Pre-existing cognitive disorders are risk factors for developing PICS.	.56	.58	.44	.97	.97
17	Prolonged ICU stay may reduce the risk of developing PICS.	.83	.58	.72	.97	.97
18	ICU patients treated with opioids and/or benzodiazepines are at low risk of developing PICS.	.59	.49	.50	.97	.97
19	Obesity may increase the risk of developing PICS.	.32	.40	.26	.97	-
20	ICU patients with a history of delirium are at risk of developing PICS.	.83	.37	.64	.97	.97
21	ICU patients with multiple organ failure are at risk of developing PICS.	.82	.33	.76	.97	.97
22	Hyperglycemia is not a risk factor affecting the development of PICS.	.54	.53	.51	.97	.97
23	Muscle weakness is not a symptom of PICS.	.62	.33	.47	.97	.97
24	Loss of motor function is a symptom of PICS.	.72	.30	.56	.97	.97
25	Weight loss is not a symptom of PICS.	.51	.30	.46	.97	.97
26	Fatigue is a symptom of PICS.	.74	.30	.62	.97	.97
27	Decreased cognitive functioning is a symptom of PICS.	.77	.44	.66	.97	.97
28	There is no relationship between memory problems and PICS.	.70	.35	.66	.97	.97
29	Brain fog (a state of cognitive impairment characterized by reduced attention, memory problems, and impaired thinking) is a symptom of PICS.	.79	.40	.72	.97	.97
30	Impaired concentration is a symptom of PICS.	.79	.30	.72	.97	.97
31	Hallucinations are not symptoms of PICS.	.57	.40	.49	.97	.97
32	Delirium is a symptom of PICS.	.71	.47	.53	.97	.97
33	Anxiety and depression are not associated with PICS.	.81	.58	.76	.97	.97
34	Post-traumatic stress disorder is not a symptom of PICS.	.64	.58	.57	.97	.97
35	PICS symptoms can persist for several months to years after discharge from the ICU.	.88	.72	.80	.97	.97
36	Feelings of guilt and anger among family members of ICU patients are major symptoms of post-intensive care syndrome-family (PICS-F).	.88	.53	.78	.97	.97
37	Family members of ICU patients may experience insomnia, decreased appetite, and severe fatigue even after the patient is discharged from the ICU.	.90	.72	.77	.97	.97
38	Spontaneous breathing trials are not one of the preventive interventions for PICS.	.59	.49	.53	.97	.97
39	Avoiding prolonged sedation is one of the preventive interventions for PICS.	.68	.67	.52	.97	.97
40	Providing adequate and balanced nutrition to ICU patients is not one of the preventive interventions for PICS.	.79	.79	.64	.97	.97
41	Late mobilization of ICU patients is one of the preventive interventions for PICS.	.73	.58	.48	.97	.97
42	Reducing environmental stressors to ensure quality sleep for ICU patients is one of the preventive interventions for PICS.	.90	.77	.86	.97	.97
43	Effective communication with ICU patients is one of the preventive interventions for PICS.	.91	.74	.86	.97	.97
44	Pain management in ICU patients is one of the preventive interventions for PICS.	.90	.63	.83	.97	.97
45	Family-centered care is not one of the preventive interventions for PICS.	.77	.53	.59	.97	.97
46	Reducing noise, adjusting lighting, and providing orientation cues with clocks and calendars are among the preventive interventions for PICS by reducing environmental stress in the ICU.	.91	.67	.86	.97	.97
47	ICU diary (a diary written by healthcare providers and family members about events that occur during the patient's ICU stay) is one of the preventive interventions for PICS.	.85	.79	.69	.97	.97
48	Cognitive therapy is not a treatment option for patients experiencing PICS.	.77	.49	.62	.97	.97
49	Physical therapy and rehabilitation are treatment options for patients experiencing PICS.	.88	.30	.76	.97	.97
50	Peer support (an activity that includes communication, empathy, and advice among patients with ICU experiences) is not an effective treatment for patients experiencing PICS.	.78	.44	.68	.97	.97
51	Psychological therapy is one of the treatment options for patients experiencing PICS.	.86	.49	.80	.97	.97
52	Nutritional management is not an effective treatment for patients experiencing PICS.	.83	.53	.72	.97	.97
53	Multidisciplinary collaboration is important for the prevention and management of PICS.	.91	.47	.79	.97	.97
54	Additional management of PICS is not necessary after ICU discharge.	.84	.40	.64	.97	.97
	Mean	.75	.50	.64		

Item No. 1-6 = General information; Item No. 7–22 = Risk factors/causes; Item No. 23 –37 = Signs/symptoms; Item No. 38–54 = Prevention/treatment interventions. Item numbers in Table 3 refer to the 54-item preliminary version. After deletion of Item 19, the remaining items were renumbered from 1 to 53 in the final K-PICS-KT presented in Appendix 1. IDI = Item difficulty index; DI = Discrimination index; CITC = Corrected item–total correlation; KR-20 = Kuder-Richardson Formula 20. ^†^Values ranged from 0.971 to 0.972, rounded to 0.97; ^‡^Values ranged from 0.971 to 0.973, rounded to 0.97

**Table 4.** Test–retest Reliability of the K-PICS-KT (N = 37)

Test score	Retest score	Intraclass correlation coefficient		
M ± SD	M ± SD	ICC	95% CI	*p*
41.24 ± 12.90	41.95 ± 12.81	.94	0.88–0.97	< .001

ICC was calculated using a two-way mixed-effects model, single measurement, and absolute agreement. K-PICS-KT = Korean version of the Post-Intensive Care Syndrome Knowledge Test; M = Mean; SD = Standard deviation; ICC = Intraclass correlation coefficient; CI = Confidence interval.
