# Supplementary material for: Cross-cultural adaptation and validation of the Korean version of the Post-Intensive Care Syndrome Knowledge Test (K-PICS-KT): a methodological study
Source: J Korean Biol Nurs Sci. 2026 Aug 27;28(3):413–27. [Article in Korean] doi: 10.7586/jkbns.26.027 (PMC13533610; doi:10.7586/jkbns.26.027)
Supplement: Supplementary file 1 [file jkbns-26-027-Appendix-1.pdf]

Appendix 1. Korean version of the Post-Intensive Care Syndrome Knowledge Test (K-PICS-KT)

다음은 집중치료 후 증후군에 대한 지식을 묻는 문항입니다. 각 문항을 자세히 읽고 '맞다', '틀리다', '모른다' 중 하나에 √ 표시 해주시기 바랍니다.

분류	번호	문항	맞다	틀리다	모른다
General information	1	집중치료 후 증후군(post-intensive care syndrome, PICS)은 환자의 중환자실 경험 후 발생하는 합병증을 의미한다.			
	2	PICS는 환자가 중환자실에 입원해 있는 동안에도 발생할 수 있다.			
	3	PICS는 중환자실에 입원한 환자의 가족에게도 발생할 수 있다.			
	4	PICS 환자는 신체적 회복 이후에도 원래의 직업이나 일상생활로 복귀하는 데 어려움을 겪을 수 있다.			
	5	섬망은 PICS와 동일한 질환이다.			
	6	PICS는 중환자실 퇴실 후 단기간에 자연 소실되는 경우가 대부분이다.			
Risk factor/ Causes	7	스트레스는 PICS 발생 위험을 높일 수 있다.			
	8	양질의 적절한 수면을 취하지 못한 중환자실 환자는 PICS 발생 위험이 있다.			
	9	장기간 부동 상태의 중환자실 환자는 PICS 발생 위험이 낮다.			
	10	중증 감염이 있는 중환자실 환자는 PICS 발생 위험이 있다.			
	11	영양 상태는 PICS 발생에 영향을 미치는 요인이 아니다.			
	12	중환자실 환자의 장기간 진정제 투여는 PICS 발생 위험을 감소시킨다.			
	13	호흡부전이 있는 중환자실 환자는 PICS 발생 위험이 있다.			
	14	고령의 중환자실 환자는 PICS 발생 위험이 낮다.			
	15	기존의 정신과적 질환은 PICS 발생 위험요인이 아니다.			
	16	기존의 인지기능 장애는 PICS 발생 위험요인이다.			
	17	중환자실 장기 입원은 PICS 발생 위험을 낮출 수 있다.			
	18	오피오이드/벤조디아제핀(opioid/benzodiazepine) 계열 약물 치료를 받은 중환자실 환자는 PICS 발생 위험이 낮다.			
	19	섬망 병력이 있는 중환자실 환자는 PICS 발생 위험이 있다.			
	20	다발성 장기 부전이 있는 중환자실 환자는 PICS 발생 위험이 있다.			
	21	고혈당은 PICS 발생에 영향을 미치는 위험요인이 아니다.			
Signs/ Symptoms	22	근력 저하(muscle weakness)는 PICS 증상이 아니다.			
	23	운동 기능 소실은 PICS 증상이다.			
	24	체중 감소는 PICS 증상이 아니다.			
	25	피로는 PICS 증상이다.			
	26	인지 기능 저하는 PICS 증상이다.			
	27	기억력 문제와 PICS는 관련이 없다.			
	28	브레인 포그(brain fog: 주의력 저하, 기억력 저하, 사고력 저하 등 인지 기능 저하 상태)는 PICS 증상이다.			
	29	집중력 저하는 PICS 증상이다.			
	30	환각은 PICS 증상이 아니다.			
	31	섬망은 PICS 증상이다.			
	32	불안과 우울은 PICS와 관련이 없다.			
	33	외상 후 스트레스 장애는 PICS 증상이 아니다.			
	34	PICS 증상은 중환자실 퇴실 후 수개월에서 수년까지 지속될 수 있다.			
	35	중환자실 환자의 가족이 느끼는 죄책감이나 분노는 집중치료 후 가족 증후군(post-intensive care syndrome-family, PICS-F)의 주요 증상 중 하나이다.			
	36	중환자실 환자의 가족은 환자 퇴원 후에도 불면증, 식욕 부진, 극심한 피로감을 느낄 수 있다.			
Prevention/ Treatment interventions	37	자발호흡 시도는 PICS를 예방할 수 있는 중재가 아니다.			
	38	장기간 진정을 피하는 것은 PICS를 예방할 수 있는 중재 중 하나이다.			
	39	중환자실 환자에게 충분하고 균형 잡힌 영양을 제공하는 것은 PICS를 예방할 수 있는 중재가 아니다.			
	40	중환자실 환자가 늦게 거동을 시작하는 것(Late mobilization)은 PICS를 예방할 수 있는 중재 중 하나이다.			

(Continued to the next page)

https://doi.org/10.7586/jkbns.26.027

Appendix 1. Continued

분류	번호	문항	맞다	틀리다	모른다
	41	중환자실 환자의 양질의 수면을 보장하기 위해 환경적 스트레스 요인을 줄이는 것은 PICS를 예방할 수 있는 중재 중 하나이다.			
	42	중환자실 환자와 원활한 의사소통은 PICS를 예방할 수 있는 중재 중 하나이다.			
	43	중환자실 환자의 통증 관리는 PICS를 예방할 수 있는 중재 중 하나이다.			
	44	가족 중심 간호는 PICS를 예방할 수 있는 중재가 아니다.			
	45	소음 감소, 조명 조절, 시계와 달력을 이용한 지남력 제공은 중환자실의 환경적 스트레스를 줄이고 PICS를 예방할 수 있는 중재 중 하나이다.			
	46	중환자실 다이어리(중환자실 입실 중 의료진과 가족이 환자에게 일어나는 일에 대해 적는 일기 형식)는 PICS를 예방할 수 있는 중재 중 하나이다.			
	47	인지 치료는 PICS 환자에게 고려할 수 있는 치료방법이 아니다.			
	48	물리치료와 재활은 PICS 환자에게 적용 가능한 치료 옵션 중 하나이다.			
	49	동료지지(중환자실 경험을 한 환자 간 소통, 공감 및 조언 제공을 포함하는 활동)는 PICS 환자에게 효과적인 치료가 아니다.			
	50	심리 치료는 PICS 환자에게 적용 가능한 치료 옵션 중 하나이다.			
	51	영양 관리는 PICS 환자에게 효과적인 치료가 아니다.			
	52	PICS 예방과 관리를 위해 다학제적 협력(의사, 간호사, 재활치료사, 영양사, 정신건강 전문가 등)이 중요하다.			
	53	중환자실 퇴실 후 PICS에 대한 추가적인 관리가 필요하지 않다.			
